# The Relation between Scores on Noise Annoyance and Noise Disturbed Sleep in a Public Health Survey

**DOI:** 10.3390/ijerph110202314

**Published:** 2014-02-21

**Authors:** Frits van den Berg, Claudia Verhagen, Daan Uitenbroek

**Affiliations:** GGD Amsterdam Public Health Service, P.O. Box 2200, Amsterdam 1000CE, The Netherlands; E-Mails: cverhagen@ggd.amsterdam.nl (C.V.); Daanuitenbroek@ggd.amsterdam.nl (D.U.)

**Keywords:** noise, annoyance, sleep disturbance, aircraft noise, road traffic noise, noise survey

## Abstract

The relation between responses to survey questions on noise annoyance and self-reported sleep disturbance has been analysed to gain insight in its dependency on noise source or noise type and on individual characteristics. The results show a high correlation between responses (scores 0–10) with Pearson’s correlation coefficient close to 0.8 for respondents who report hearing the source. At the same level of annoyance, scooters and neighbours are associated with more sleep disturbance, air and road traffic with less. The relation between Annoyance (A) and Sleep Disturbance (SD) is also significantly related to age, the use of sleeping drugs, and living alone. However, the differences in the A-SD relations with respect to source and characteristic are small. Noise-related sleep disturbance is associated more strongly to noise annoyance than it is to noise exposure. For transportation noise both scores are more often equal when the annoyance score is 7 or higher; this change in scoring behaviour could be an indication for a change to severe annoyance.

## 1. Introducton

Noise annoyance and sleep disturbance from noise are the most prevalent effects of residential noise exposure [1]. Both effects can be assessed in population surveys using standard questions to gauge self-reported noise annoyance and sleep disturbance from noise.

Noise annoyance and sleep disturbance have been investigated extensively. The World Health Organization has published overviews of research results in 1999 and 2009 [1,2]. Most of the literature pertains to the relation of annoyance or sleep disturbance with noise level and mostly concerns transportation noise. Relations with the level of transportation noise have been published for both effects [3,4].

Early on it has been pointed out that self-reported noise-induced sleep disturbance could also be a matter of attribution (being awakened by whatever cause is attributed to a noise source), not of actually being awakened by that source [5]. Subjective self-reports on sleep quality or awakenings do not correlate well with more objective measures of sleep disturbance [6,7]. Nevertheless, (noise-induced) sleep disturbance is related to noise level [4], though the number of loud noise events may be more important than an average noise level [8,9]. There is some evidence that noise-related sleep disturbance may be more closely related to noise annoyance than to noise level [10]. The presence and degree of noise annoyance or noise-related sleep-disturbance depends on acoustical and non-acoustical characteristics. For example, noise events could have more effect than continuous noise at the same average sound level [11,12,13]. Also, meaningful sounds (from people, music) could be more disturbing than meaningless or more neutral sound as from transportation [14].

Personal factors such as age, gender, occupational class, state of health and loneliness [4,5,15] may contribute to disturbed sleep, including cases when noise is the alleged cause. Also, an association was found between disturbed sleep and (neuro-) psychological problems or distress, but it was not clear if the use of sleeping drugs or tranquilizers was directly related to noise-induced sleep disturbance [16].

In this study, we investigated the correlation between responses to noise annoyance and sleep disturbance. The original reason to investigate this was very practical: if there is a high correlation, the use of only one of these questions in a survey would be sufficient. This would leave room for another question or could shorten the questionnaire. Based on earlier research results two more topics were investigated:

- does the relation between responses to questions on noise annoyance and sleep disturbance depend on the sound source or type of sound, such as intermittent or meaningful sound?
- does this relation depend on personal factors such as age, distress or use of sleeping drugs?

Data are available from a general health survey that was not specifically designed for the purpose of this study, but it did provide relevant data for our purpose.

The research questions involve associations between subjective responses and as such address perceived sound sources but not sound levels. Although the risk of annoyance depends on the noise level, annoyance as such and its effects may be similar at whatever noise level has triggered an annoyance reaction. According to the WHO [2] both objective (sound level) and subjective (annoyance) ‘exposures’ may serve as independent exposure variables in statistical analyses of noise and health end points.

## 2. Methods

### 2.1. Study Group

**Figure 1:**
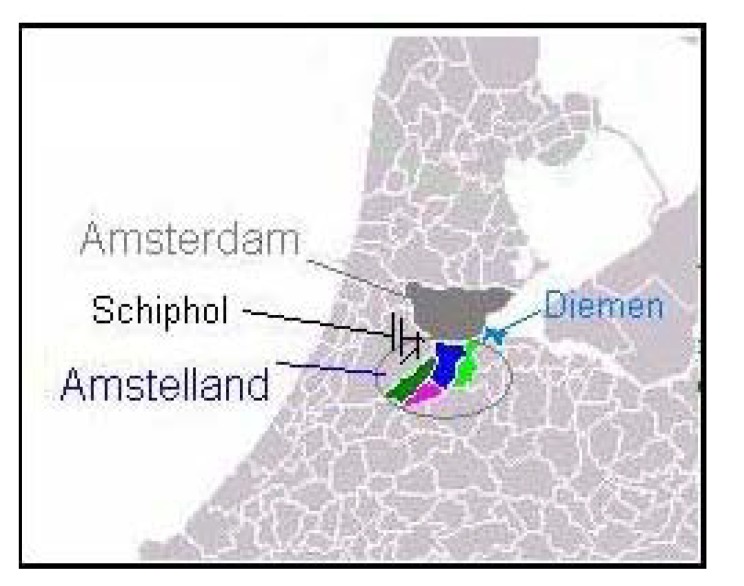
Map of west of the Netherlands with survey area and airport.

The Dutch regional and local Public Health Services (PHS, in Dutch: GGD) regularly survey the status of health and health related topics of the population in their area. The questions in the surveys have been standardized in the last decade, but PHSs are free to choose which questions to include in their surveys. With respect to environmental issues a question on noise annoyance is included by most, if not all PHSs; a question on sleep disturbance related to noise is included by fewer PHSs. The survey in our analysis included both questions and was held in 2010 in five municipalities just south of the city of Amsterdam. Together with Amsterdam these municipalities form the work area of GGD Amsterdam. Most of the population, comprising 173,000 people, lives in urban area contiguous with the city of Amsterdam and close to the busy airport of Schiphol: see Figure 1. 6,876 persons, distributed over five age groups and randomly selected from the population administrations, were asked to take part in the survey. 3,817 persons (response rate 55.5%) completed the paper questionnaire or an online questionnaire (Table 1).

**Table 1:** Number of respondents per age group and percentage of male respondents.

Age group	19–34	35–49	50–64	65–74	75+	All
response rate	34%	45%	63%	77%	65%	56%
# respondents	570	584	814	999	850	3817
% male	39%	42%	47%	48%	43%	44%

There was no non-response test. Respondents as a group did not deviate substantially from the Dutch population with respect to ethnicity, marital or employment status. No national data were available on education level that could be compared to the study group.

### 2.2. Noise Questions in the Questionnaire

The question on noise annoyance was the question standardized in ISO/TS-15666 [17]. The question on noise-induced sleep disturbance has been modelled after the standard annoyance question, but has not been standardized itself by the International Organization for Standardization.

The question on annoyance is formulated as:

“ Below is a scale from 0 to 10 where you can indicate to what degree you are annoyed, disturbed or irritated when you are at home. If you are not annoyed at all, please tick a 0, if you are extremely annoyed, please tick a 10. If you are somewhere in between, please tick a number between 0 and 10. If the sound is not present in your home, you can tick this in the last column.

Thinking about the past 12 months, which number from 0 to 10 indicates best to what degree you are annoyed, disturbed or irritated from sound from sources listed below when you are at home? Please put a tick on every line.”

The question on sleep disturbance is formulated as:

“To what degree is your sleep disturbed when you are at home from sound from sources listed below? Think about the situation in the past 12 months. Please tick every line.”

Below each question is a list of noise sources:

- Traffic on roads with a speed limit higher than 50 km/h (‘highway traffic’)
- Traffic on roads with a speed limit of 50 km/h or less (‘city traffic’)
- Trains
- Urban or regional tram and metro (‘trams’)
- Aircraft
- Schiphol airport (taxiing, engine testing and/or other ground activities) (‘airport’)
- Industry (includes smaller and bigger businesses and factories)
- Neighbours
- Mopeds or scooters with and without licence (‘scooters’)
- Other (not reported here).

In each question the respondent could tick a separate box labeled ‘Not audible’ preceding the eleven point scale. Both questions together filled a complete page of the questionnaire. In the remainder of this article the names of the noise sources will be taken from the list above; if a short name is mentioned between quotation marks, this name will be used.

### 2.3. Characteristics of Noise Source

Of course, an important cause for noise-induced sleep disturbance is the extent to which the noise source is audible at night. In general transportation sources in the Netherlands are less noisy at night and often the night time penalty of 10 dB balances the reduction in nocturnal noise production when compared to daytime values. It is expected that scooters and the airport will have a similar diurnal pattern. The night time reduction is less for highways and busy railroads, but night time noise levels will be lower than daytime levels. Neighbour noise is a main source of self-reported annoyance and sleep disturbance, in the Netherlands second only to road traffic noise.

Even when sources have the same temporal characteristics they may be different in spectral composition. When compared to high frequency sound, low frequency sound of the same outdoor level will cause higher indoor levels because of the reduced façade insulation at low frequencies. This again may be influenced by windows: when sleeping with an open window façade insulation is less important. Dwelling characteristics (single or double glazing, insulation) and residents’ behaviour (e.g., by having windows open, ajar or closed, sleeping with ear plugs, or sleeping on the quiet side) can influence indoor exposure from outdoor sources. Such effects probably cause variations in annoyance and sleep disturbance, even within areas of similar building types and practices. In a survey an average outcome of such variations is effectively measured.

### 2.4. Individual Characteristics in the Questionnaire

The response on both noise questions can be investigated for a number of individual characteristics addressed in the survey questionnaire. Characteristics that were thought to be relevant for this analysis, each stratified into the two categories mentioned between brackets, are:

- Age (19–64, 65+)
- Gender (male, female)
- Perceived health (excellent/good, moderate/bad)
- Use of sleeping drugs (high = at least once every two weeks, low = less)
- Risk to suffer from anxiety/depression (high, medium/low)
- Feeling lonely (high, low)
- Satisfied with house (scale 1–10: yes = score 6–10, no = 1–5)
- Satisfied with living environment/neighbour-hood (scale 1–10: yes = score 6–10, no = 1–5)
- Living alone (yes, no)

### 2.5. Analysis

The survey results were used to investigate the relation between response scores for the noise annoyance and sleep disturbance questions. For each noise source Pearson’s correlation coefficient was calculated for the relation between annoyance score A and sleep disturbance score SD. The relations were analysed either according to annoyance score units (ranging from 0 to 10), or according to a range of annoyance scores: highly annoyed (HA) means a score of 8, 9 or 10, annoyed (but not highly) means a score of 4, 5, 6 or 7. Sleep disturbance was classified as highly sleep disturbed (HSD, scores 8, 9 or 10) or sleep disturbed (but not highly) (SD; scores 4, 5, 6 or 7). The classifications are similar to those given by Schultz (highly: upper three scales of eleven-point scale or upper two of seven-point scale, *i.e.*, upper 27%–29% of scale) and close to the classification by Miedema and Oudshoorn (highly: upper 28% of scale; (at least) a little annoyed: lower 28% of scale excluded) [18,19]. In the present study we investigated the response scores as such and we used the classification of Schultz for HA and HSD (upper three scales of eleven-point scale). For annoyance we used a lower boundary of 4 because that scale is halfway between nil and the high annoyance lower boundary. We have not used the classifications of Miedema and Oudshoorn as they would lead to the same responses being divided over different annoyance ranges for responses near class boundaries.

**Figure 2:**
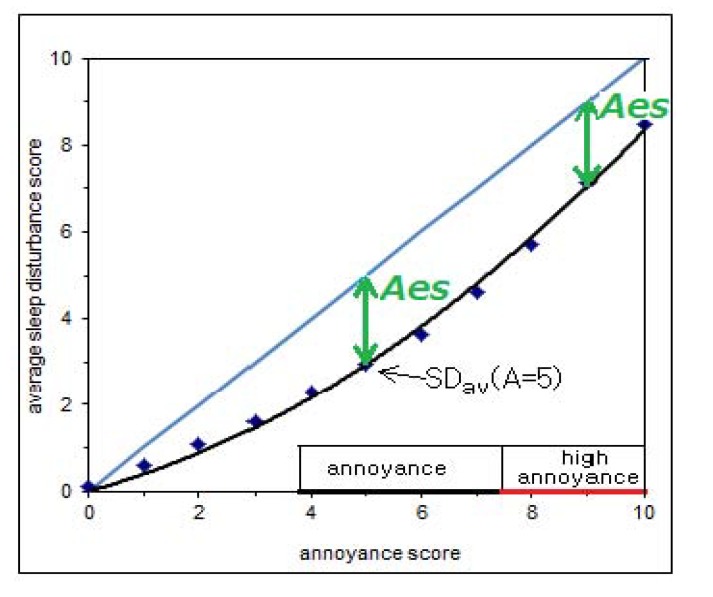
Relation between average sleep disturbance score (SD_av_) and annoyance score; illustrates annoyance ranges and Annoyance excess score *Aes.*

For each noise source and for each A the SD averaged over all respondents (SD_av_) can be calculated. The difference between the score on annoyance and the average sleep disturbance (A − SD_av_) or the *Annoyance excess score* or *Aes* was used to compare sources. This is illustrated in Figure 2: the vertical difference between the data points (SD_av_) and the diagonal (SD = A) is the *Aes*. If SD_av_ is determined over the range of (high) annoyance, *Aes* is determined from the average over all SD scores and the average annoyance score in that range.

To determine whether differences between sources are significant, the differences were tested according to Pearson & Filon as two correlations based on four different variables from the same sample, using the number of respondents and the six correlations between the four variables [20]. Differences between sources can be quantified as a difference in SD_av_ or *Aes*.

To investigate differences between groups stratified according to individual characteristics again *Aes* was used. In fact, the analysis shows for which noise sources and individual characteristics there is a relatively low sleep disturbance score, *i.e.*, a bigger value of *Aes*, and for which sources or characteristics the sleep disturbance score is relatively high, *i.e.*, *Aes* has a low value. Differences with a probability (or *p*-value) lower than 0.05 are considered statistically significant.

All analyses have been performed for only those respondents that reported hearing a noise source. This is in contrast to the determination of prevalence of noise annoyance or noise-induced sleep disturbance in the population, where also respondents that do not hear a source must be included. This study aims to analyse associations between both effects and to this end respondents not hearing a source are not relevant.

## 3. Results

### 3.1. Results with Respect to Noise Source

Table 2 gives an overview of the number of respondents (out of a total of 3817) that reported hearing noise from a specific source and the percentages of those that report to be annoyed (%A, 4 ≤ A < 8) or highly annoyed (%HA, A ≥ 8), or sleep disturbed (%SD, 4 ≤ SD < 8) or highly sleep disturbed (%HSD, SD ≥ 8) by each source. Also shown in Table 2 are the Pearson’s correlations of the individual scores. All correlation coefficients have a value between 0.74 and 0.84 and are significant at *p* = 0.01 (2-tailed).

**Figure 3:**
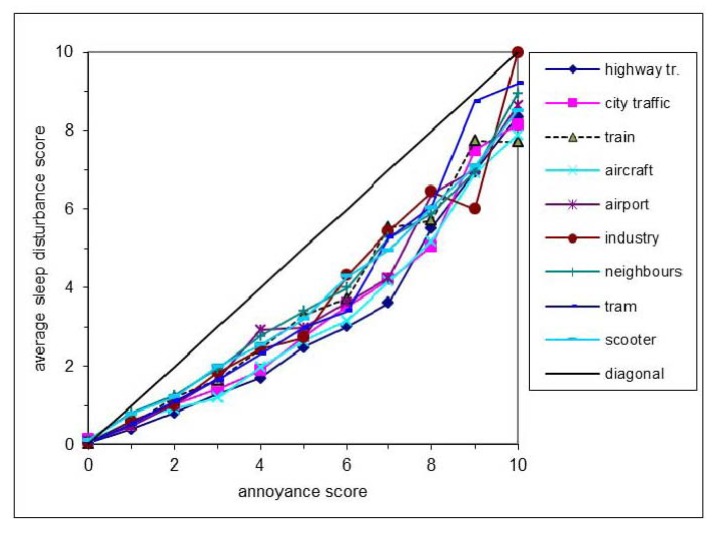
Relation between average sleep disturbance and annoyance scores per noise source.

Figure 3 shows how the average sleep disturbance score changes for each source when the annoyance score increases from 0 to 10. Data points based on 20 respondents or less (occurring at annoyance scores of 7 and/or higher for trains, trams and industry) are excluded. Every line in Figure 3 can be approximated by a quadratic fit with respect to the average SD scores (SD_av_). The best fit to all scores (all sources together) is SD_av_ = 0.044∙A^2^ + 0.39∙A (in fact, the data points and fit plotted in Figure 2 are those for all sources from the survey). For each separate source the best quadratic fit (least squares method) to SD_av_ at first increases more slowly than A (slope at A = 0 is 0.1 to 0.5, depending on noise source) and finally increases faster than A (slope at A = 10 is 1.1 to 1.6). The change-over from a slower to a faster increase of the best fit is where the slope equals 1; for all sources together this is at A = 6.6, for individual sources this varies from 5.4 to 7.8.

**Table 2:** Correlation between individual scores on annoyance (A) and sleep disturbance (SD); number of respondents that hear source, and percentages of those who hear source that are (highly) annoyed (%(H)A) and (highly) sleep disturbed (%(H)SD). Ordered by percentage of respondents highly annoyed.

Noise source	# Respondents that hear source	Corr. coeff. SD *vs*. A	%A (4 ≤ A < 8)	%HA (8 ≤ A)	%SD (4 ≤ SD < 8)	%HSD (8 ≤ SD)
Aircraft	2,589	0.76	34%	22%	22%	11%
Scooters	2,326	0.80	27%	12%	18%	8%
Airport	1,101	0.83	21%	10%	14%	7%
City traffic (≤50 km/h)	1,972	0.74	22%	7%	12%	4%
Neighbours	2,137	0.80	19%	7%	13%	5%
Highway traffic (>50 km/h)	1,451	0.75	24%	7%	11%	4%
Trains	794	0.84	14%	5%	9%	2%
Industry/businesses	792	0.83	10%	3%	6%	3%
Trams/metro	856	0.82	10%	3%	6%	2%

Depending on the noise source 4% to 12% of respondents that hear that source give an SD score in excess of the A score, usually with little difference between both scores (*i.e.*, a low negative value of *Aes*). Of these, the highest percentages occur for noise from industry (12%) and neighbours (11%), lowest for airplanes (4%) and highway traffic (5%). Relatively high SD scores (SD > A + 2) are negligible in numbers and for every single source occur for less than 2% of the respondents. Figure 3 shows that for some sources the relation SD *vs*. A is closer to the diagonal than for other sources, implying that for those sources the risk of sleep disturbance is higher at the same value of A. We consider a sleep disturbance score as being close to the diagonal if it differs 2 units or less from the annoyance score (|A − SD| = |*Aes*| ≤ 2). Thus, *Aes* > 2 implies a relatively low SD score.

Table 3 shows results for respondents that could hear a source and were either annoyed or highly annoyed. Here noise sources are ordered according to their average sleep disturbance score. For noise from industry, tram and trains there were less than 100 respondents in the annoyance range concerned (trains only in high annoyance range). A higher average Annoyance excess score *Aes*, with a higher value indicating less similar scores on A and SD, corresponds to a lower SD_av_ score. In fact, SD_av_ + *Aes* should equal 5.5 in the annoyance range and 9.0 in the high annoyance range if there were the same number of respondents at each annoyance score. As the distribution of respondents over the annoyance scale is somewhat skewed, SD_av_ + *Aes* is close to, but not always exactly equal to 5.5 (4 ≤ A < 8) or 9.0 (8 ≤ A).

**Table 3:** Scoring characteristics of respondents that can hear source, separated in two annoyance ranges (annoyed and highly annoyed). Shown for each range: percentage of respondents (highly) annoyed (%(H)A), average score difference A − SD_av_ (= *Aes*) and average sleep disturbance score (SD). Ordered according to average SD score, separately for both annoyance ranges.

	Annoyed (4 ≤ A < 8)				Highly annoyed (8 ≤ A)			
Noise source	%A	Average *Aes*	Average SD		Noise source	%HA	Average *Aes*	Average SD
Scooters	27%	1.8	3.7		Tram	9%	1.4	7.2
Neighbours	19%	1.6	3.6		Airport	10%	1.6	7.2
Industry	10%	1.8	3.6		Industry	3%	1.5	7.0
Airport	21%	2.1	3.5		Neighbours	7%	1.8	7.0
Trains	14%	1.8	3.5		Scooters	12%	1.8	6.9
Tram	21%	2.0	3.2		Trains	5%	2.1	6.6
City traffic	22%	2.4	3.1		City traffic	7%	2.2	6.6
Aircraft	34%	2.5	3.0		Aircraft	22%	2.4	6.4
Highway traffic	24%	2.8	2.7		Highway traffic	7%	2.5	6.1

According to Table 3 scooters, neighbours, industry and the airport are associated to higher sleep disturbance scores compared to (city and highway) road traffic and aircraft in both annoyance ranges. Trains have a position between these noise source groups and trams have a different position in the annoyance and high annoyance range. Whether the relations between A and SD are significantly different between sources can be calculated from the correlation coefficients between all pairs of variables (A and SD from sources 1 and 2) [20]. For pairs of sources where the relations between A and SD differ significantly from each other, the difference in average SD scores for both the annoyance range and high annoyance range (yellow) is given in Table 4. Because of the large number of possible correlations, relations are considered to be significant here when *p* < 0.01. Where values are shown in Table 4, in fact *p* < 0.0001 (two-sided).

**Table 4:** Differences in average SD score per annoyance range between pairs of noise sources; differences are given when significant and separately in annoyance (4 ≤ A < 8, above diagonal) and high annoyance range (A ≥ 8; yellow below diagonal); SD scores of sources on top line are subtracted from sources in left column. x = diagonal of table.

	Scooters	Neighbours	Industry	Airport	Trains	Tram	City traffic	Aircraft	Highway traffic
Scooters	x		0.10	0.21	0.23	0.52		0.67	0.99
Neighbours		x		0.17	0.19	0.48		0.63	0.95
Industry	0.15		x	0.11	0.13	0.42		0.57	0.89
Airport	0.30	0.17	0.15	x			0.40	0.46	
Trains	−0.33	−0.46	−0.48		x		0.38	0.44	
Tram	0.33	0.20	0.18			x	0.09	0.15	
City traffic				−1.09	−0.46	−1.12	x	0.06	0.38
Aircraft	−0.50	−0.63	−0.65	−0.80	−0.17	−0.83	0.29	x	0.32
Highway traffic	−0.34	−0.47	−0.49				0.45	0.16	x

**Figure 4:**
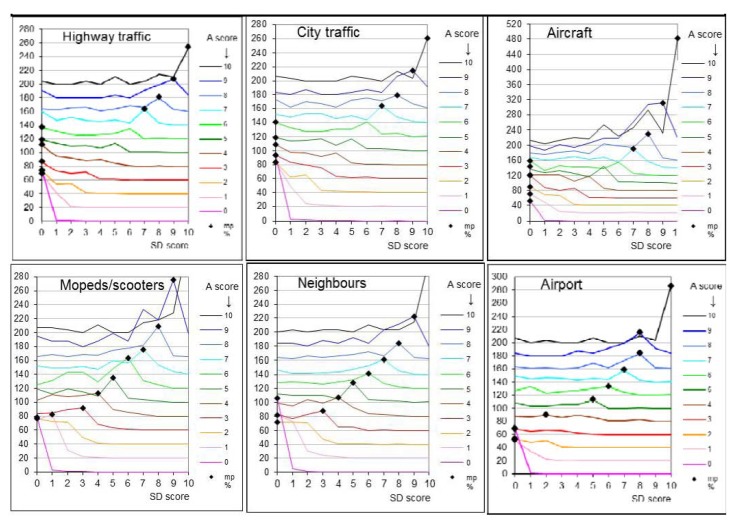
distribution of Sleep Disturbance scores score given by respondents that report to hear the sources in top of graphs; abscissa shows % of SD scores at each SD/A score, distributions shown for each Annoyance score (A) separately and shifted upwards with +A∙20%; diamonds mark most prevalent choice at each A (most prevalent % or mp%).

The scoring behaviour is such that at low annoyance scores the most prevalent choice for sleep disturbance from road or air traffic is 0, but when the annoyance score exceeds a value of 6 the most prevalent choice for sleep disturbance is a score equal to the annoyance score (Figure 4). For scooters the most prevalent SD score is equal to the annoyance score over the entire range of A scores. For neighbours’ and airport noise the distributions are intermediate (other noise sources are not shown in Figure 4 because at higher A scores there are less than 20 respondents at each A score).

### 3.2. Influence of Individual Characteristics

The responses to both noise-related questions can be analysed with respect to a number of individual characteristics of respondents. This was investigated for aircraft and road traffic. Table 5 gives an overview of the characteristics for which significant *Aes* differences occur (see Section 2.4 for a description of groups) for all respondents that hear the source at hand. A negative sign in *Aes* differences in Table 5 means that the group with the characteristic mentioned first has a higher risk on sleep disturbance (or: is closer to the diagonal in Figure 2) at the same annoyance score.

**Table 5:** Differences in *Annoyance excess score* (*Aes*) in relation to individual characteristics. Values shown only when difference is significant (*p* < 0.05).

Noise type → Characteristic	Highway Traffic	City Traffic	Aircraft
	*Aes* (= A − SD_av_)		
Gender (male-female)			0.2
Age (19/64–65+)	0.8	0.5	0.5
Perceived health (good-bad)			0.4
Use of sleeping drugs (high-low)	−0.5	−0.4	−0.5
Living alone (yes-no)	−0.3		−0.4

Some individual characteristics have a significant effect on annoyance as well as on sleep disturbance. If the effects are similar in magnitude, this will not result in a change in *Aes*. House and neighbourhood satisfaction both lead to a lower risk on annoyance and on sleep disturbance; a high anxiety risk is associated with a higher risk on annoyance and on sleep disturbance. In these cases the net result for the difference between both, *Aes*, is not significant. Graphically, this corresponds to gliding along the SD_av_(A) curve (Figure 2), which has no influence on *Aes* (for A ≥ 4) although SD and A do change. Between age groups there is a larger difference in A than in SD and there is a net result on *Aes*. *Aes* is significantly greater for adults younger than 65 years, which is due to more annoyance in the group younger than 65. It is significantly lower for those that use sleeping drugs, due to more sleep disturbance in the group using sleeping drugs. Additionally, for aircraft noise *Aes* is significantly greater (*i.e.*, lower SD at constant A) for men, those that feel healthy, and (also for highway traffic noise) those that do not live alone. These differences are related to, respectively, more annoyance for male respondents, less sleep disturbance for those that feel healthy and both effects for those not living alone.

## 4. Discussion

In the survey results reported here, correlations between scores on annoyance and sleep disturbance are high. We used Pearson’s *r* to measure the correlation between annoyance and sleep disturbance. Pearson’s *r* is not a perfect measure for this case [21]. However, as Figure 3 shows, there is a good linear relationship near the diagonal and it can therefore be assumed that Pearson’s *r* is reliable. For our analysis this had the advantage that it allows for easy comparison between measures. Our results show that for a general (environmental) health survey it may be sufficient to predict the distribution of scores for sleep disturbance SD from the distribution of the annoyance scores A (or, *vice versa*, an average A from SD). Of course, there can be specific reasons to ask both, e.g., when investigating the effect on night shift workers or to evaluate night time noise mitigation measures.

The present results may not apply to other areas. In order to determine whether the present results are area-specific, a similar analysis has been applied to another Dutch survey in an area less urbanized and more distant to Schiphol airport [22]. The values of the correlation coefficients of the A-SD relations and the positions of the curves were similar to those found in this study.

Miedema and Vos [3,19] and Schultz [18] defined the upper 28% of the annoyance scale or score 8, 9 or 10 on an eleven point scale (Section 3.5) as serious annoyance. The present study shows a change in scoring behaviour for road and air traffic at annoyance score 7: for lower scores the most prevalent choice was SD = 0, but for A ≥ 7 the most prevalent choice was SD = A. A better criterion for high annoyance therefore may be a score of 7 or higher as in this range sleep disturbance is more often an added effect of the same intensity. Perhaps it is the added effect of perceived sleep disturbance that makes annoyance a serious health effect. The similarity of both questions (especially when placed next to each other) may have suggested a similarity in response. However, this would not explain the differences between sources.

The difference between day and night noise level apparently is not a dominant reason for the differences in *Annoyance excess score* (*Aes* = A − SD_av_). At a given annoyance score, the value of SD_av_ of highway traffic is lower (*Aes* higher) than for city traffic although highway traffic does not subside as much at night. In contrast, city traffic, tram and scooter noise are expected to follow the same diurnal variation, but they have quite different values of *Aes* or SD_av_. The results show that the value of *Aes* is related to scoring behaviour: at high *Aes* (road traffic and aircraft) sleep disturbance was reported less often at low annoyance scores whereas at low *Aes* (neighbours and scooters) sleep disturbance was reported more often also at low levels of annoyance.

There is no clear, consistent difference between intermittent and continuous sources, that is: sources that occur as separate and discrete events and sources where discrete events do not exist or where events overlap. Aircraft and scooters are clearly intermittent sources, but are very different with respect to SD_av_. Perhaps noise from the sources with a high value of SD_av_ (neighbours, scooters, industry, airport) is more erratic and therefore may attract more attention than noise from the more constant and/or predictable aircraft and road traffic and, to a lesser degree, trains. The results may support the conclusion of Kuwano *et al*. [14] that meaningful noise disturbs sleep more than meaningless or neutral noise. A related possibility is that the attitude towards the source mediates the effect, as has been shown, e.g*.*, for wind turbines [22] and church bells [23]. In the same vein, noise can also be perceived as unnecessary and then convey the meaning of inconsiderateness or even malignancy. Of course, both qualities ‑intermittency and meaning- can have separate effects on the sleep disturbance score and hence have additive effects.

To be able to report noise-induced sleep disturbance, a respondent must have been awake and at that time hear a noise. The present results show that this is apparently strongly correlated to general (daytime) noise annoyance. This could of course be due to the same noise source being present at day and night time. It could also be due to a transfer of annoyance experienced at daytime to the moment that one awakes involuntarily. This would support the statement by Langdon and Buller [5] that there is a tendency to attribute sleep disturbance to noise. This tendency could explain the higher SD scores attributed to noise by those that do not sleep well or use sleeping drugs. Also, when there is another reason for sleep disturbance (pain/discomfort at older age—as suggested in [5]—a bed partner when not living alone) this reduces the attribution to noise and thus can lead to a lower sleep disturbance score and higher *Aes*, as found in this study. 

For each noise source, correlation coefficients between annoyance and sleep disturbance are approximately 0.8 and therefore the noise annoyance scores explain over 50% of the variance in sleep disturbance scores. In contrast, noise levels explain less than 10% of self-reported sleep disturbance [4]. Annoyance thus may be a better predictor of noise-induced sleep disturbance than noise level is, confirming results from Fyhri and Aasvang [10] and the WHO statement that annoyance can be regarded as a (subjective) exposure.

Miedema and Vos [4] could not offer a plausible explanation for the ‘curvilinear’ influence of age on self-reported sleep disturbance (less SD for younger and older adults, more SD for people in their 50s). We think that, given the strong correlation between annoyance and sleep disturbance as found in this study, the curvilinear behaviour in sleep disturbance is a reflection of the same behaviour in noise annoyance [25]. To gauge sleep disturbance without suggesting respondents that noise is the cause (which could be a stimulus to attribute disturbance to noise), it may be preferable to use other instruments such as the Epworth Sleepiness Scale or Pittsburgh Sleep Quality Index in a shortened form [26]. Alternatively, questions with regard to sleep quality could be specified, e.g., by enquiring about frequency of awakenings, problems falling asleep or early awakenings related to noise.

## 5. Conclusion

This study shows that the response to noise annoyance and noise-induced/attributed sleep disturbance questions are highly correlated and therefore in a general health questionnaire one question will usually be redundant. Self-reported noise-related sleep disturbance is associated more strongly to noise annoyance than it is to noise exposure.

Our results indicate that serious annoyance from transportation noise may be better defined as an annoyance score of 7 or higher. Perhaps the added effect of sleep disturbance helps transform annoyance into a health hazard.

Clearly, noise annoyance and sleep disturbance from noise, as measured in questionnaires, are not independent. Self-reported sleep disturbance may not be very different from self-reported noise annoyance, except that it concerns the shorter night time period. The two effects probably share a common notion considering all relations between both effects show the same trend and are quantitatively very similar for all noise sources. As yet it is not clear what it is that we gauge with the question on sleep disturbance.
